# 4-(6-Benzyl-7-oxo-1,2,3,4,5,5a,5a^1^,6,7,7a,8,12b-dodeca­hydro­benzo[*f*]cyclo­octa­[*cd*]isoindol-8-yl)benzonitrile

**DOI:** 10.1107/S1600536813006880

**Published:** 2013-03-16

**Authors:** Yu-Long Zhang, Yi-Min Hu

**Affiliations:** a School of Chemistry and Materials Science, Anhui Normal University, Wuhu, Anhui 241000, People’s Republic of China

## Abstract

In the title compound, C_31_H_30_N_2_O, the *cis*-fused cyclo­hexene and cyclo­octane rings adopt boat and boat-chair conformations, respectively. The essentially planar five-membered *N*-heterocyclic ring [r.m.s. deviation = 0.017 (1) Å] is *cis*- and *trans*-fused, respectively, with the cyclo­hexene and cyclo­octane rings. In the crystal, mol­ecules are linked into inversion dimers through pairs of weak C—H⋯O inter­actions.

## Related literature

For the atom economy and environmental benefits of using a domino reaction to construct a structurally complicated mol­ecule, see: Zhao *et al.* (2012 ▶). For palladium-catalysed coupling reactions, see: Hu *et al.* (2009 ▶, 2010 ▶). For the use of condensed heterocyclic compounds as synthetic building blocks, pharmacophores and electroluminescent materials, see: Rixson *et al.* (2012 ▶). For reactions of aryl halides with olefins, see: Yu & Hu (2012 ▶); Wang & Hu (2011 ▶).
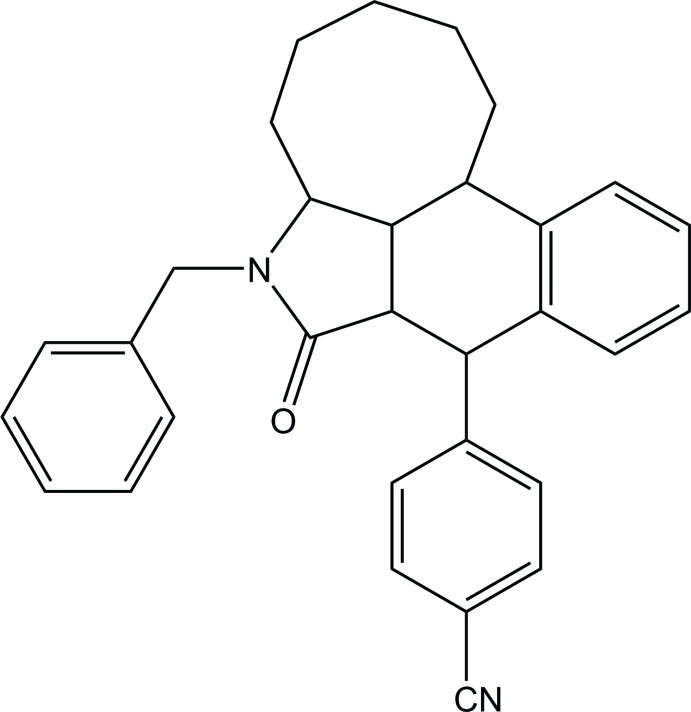


## Experimental

### 

#### Crystal data

C_31_H_30_N_2_O
*M* *_r_* = 446.57
Monoclinic, 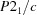

*a* = 10.0270 (11) Å
*b* = 11.2196 (13) Å
*c* = 21.445 (2) Å
β = 102.912 (1)°
*V* = 2351.5 (5) Å^3^
*Z* = 4
Mo *K*α radiation
μ = 0.08 mm^−1^
*T* = 291 K
0.26 × 0.22 × 0.20 mm

#### Data collection

Bruker SMART APEX CCD diffractometer
Absorption correction: multi-scan (*SADABS*; Bruker, 2000 ▶) *T*_min_ = 0.981, *T*_max_ = 0.985
19941 measured reflections
5413 independent reflections
4118 reflections with *I* > 2σ(*I*)
*R*_int_ = 0.024

#### Refinement

*R*[*F* ^2^ > 2σ(*F* ^2^)] = 0.046
*wR*(*F* ^2^) = 0.128
*S* = 1.04
5413 reflections
308 parameters
H-atom parameters constrained
Δρ_max_ = 0.23 e Å^−3^
Δρ_min_ = −0.18 e Å^−3^

### 

Data collection: *SMART* (Bruker, 2000 ▶); cell refinement: *SAINT* (Bruker, 2000 ▶); data reduction: *SAINT*; program(s) used to solve structure: *SHELXS97* (Sheldrick, 2008 ▶); program(s) used to refine structure: *SHELXL97* (Sheldrick, 2008 ▶); molecular graphics: *SHELXTL* (Sheldrick, 2008 ▶); software used to prepare material for publication: *SHELXTL*.

## Supplementary Material

Crystal structure: contains datablock(s) global, I. DOI: 10.1107/S1600536813006880/is5255sup1.cif

Structure factors: contains datablock(s) I. DOI: 10.1107/S1600536813006880/is5255Isup2.hkl

Supplementary material file. DOI: 10.1107/S1600536813006880/is5255Isup3.cml

Additional supplementary materials:  crystallographic information; 3D view; checkCIF report

## Figures and Tables

**Table 1:** Hydrogen-bond geometry (Å, °)

*D*—H⋯*A*	*D*—H	H⋯*A*	*D*⋯*A*	*D*—H⋯*A*
C29—H29⋯O1^i^	0.93	2.58	3.301 (2)	134

Symmetry code: (i) 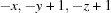.

**Fig. 1.**
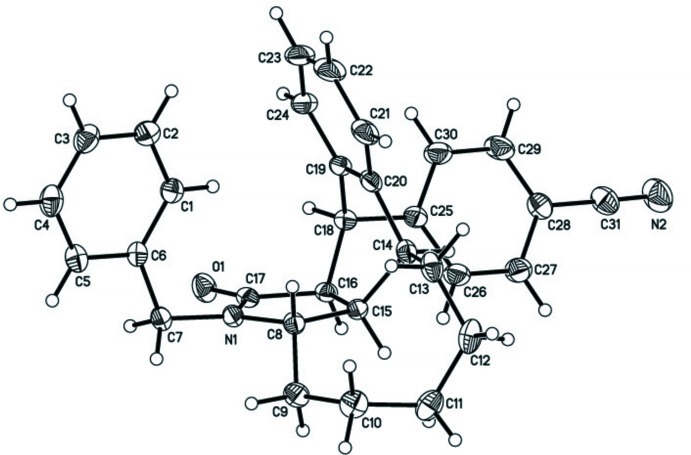
The molecular structure of the title compound, showing the atom-numbering scheme and displacement ellipsoids drawn at 30% probability level.

**Fig. 2.**
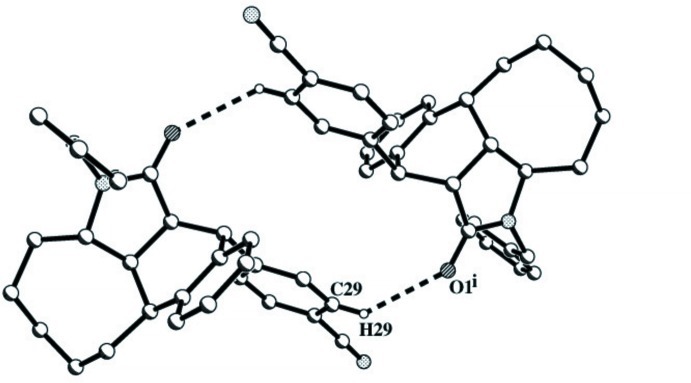
A view of a pair of racemic molecules linked by C—H···O hydrogen bonds shown as dashed lines [symmetry code (i): -x, 1 - y,1 - z].

**Fig. 3.**
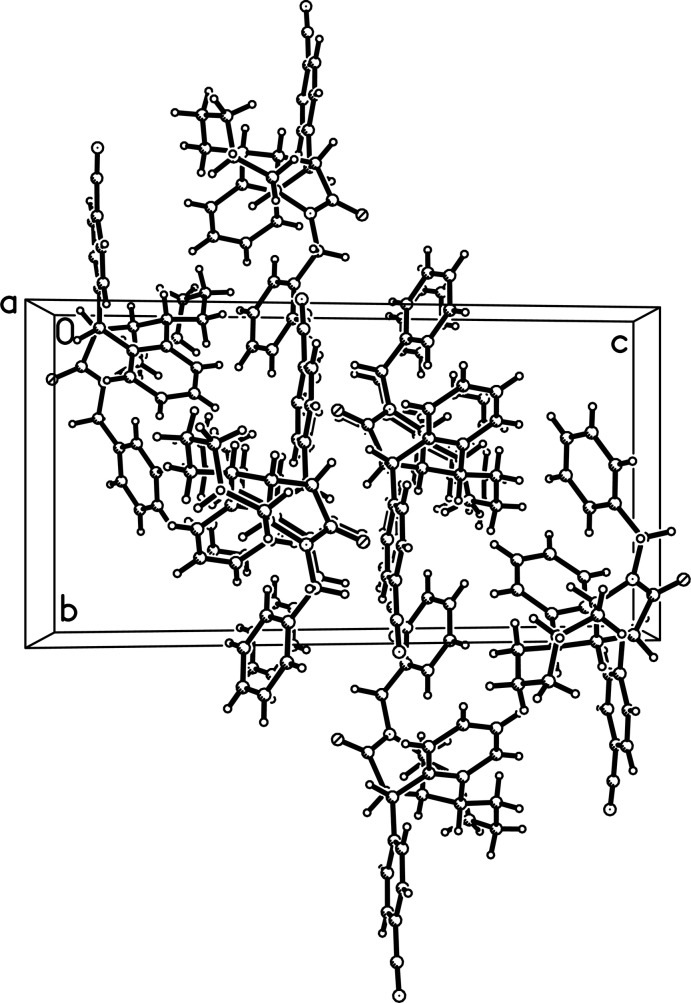
A view of the cell packing down the a axis.

## supplementary crystallographic information

### Comment

Domino reaction as an efficient protocol to construct structurally complicate molecule due to high atom economy and environmental benefits (Zhao *et al.*, 2012). Palladium-catalyzed domino reactions have become an important tool of modern organic synthesis chemistry (Hu *et al.*, 2009, 2010). Condensed heterocyclic compounds are playing increasingly important roles as synthetic building blocks, pharmacophores, and electroluminescence materials (Rixson *et al.*, 2012). We have reported some novel intermolecular and intramolecular reactions of aryl halides with the olefins (Yu *et al.*, 2012; Wang *et al.*, 2011). The reaction of 4-bromobenzonitrile with *N*-benzyl-*N*-(cyclooct-2-en-1-yl) cinnamamide, in the presence of palladium(II) acetate and triphenylphosphine, in DMF at 423 K for 18 h, gave the unexpected title product.

The title compound, C_31_H_30_N_2_O, contains three phenyl ring, one five-numbered N-heterocyclic ring, one six-membered carbon ring with a boat conformation, and one eight-memmbered carbon ring with a boat-chair conformation. All the rings are not coplanar (Fig. 1). In the molecule there are five chiral carbon atoms, C8, C14, C15, C16 and C18, but the crystal is a racemic system due to lacking of the chiral separation. In the crystal packing, there are weak intermolecular C—H···O interactions, C29—H29···O1^i^ (i: -*x*, 1 - *y*, 1 - *z*), through which dimers were formed between paired enantiomers (Fig. 2).

### Experimental

An oven-dried Schlenk flask was evacuated, filled with nitrogen, and then charged with *N*-benzyl-*N*-(cyclooct-2-en-1-yl)cinnamamide (3.45 g, 10 mmol), ethyl 4-bromobenzonitrile (2.01 g, 11 mmol), tributylamine (3 ml), PPh_3_ (52.5 mg, 0.2 mmol), Pd(OAc)_2_ (24 mg, 0.1 mol), and DMF (10 ml) to give a yellow solution. The reaction mixture was heated at 423 K with stirring. The reaction mixture was cooled to room temperature after 18 h and the resultant yellow-orange mixture was diluted with Et_2_O (10 ml). The mixture was washed with H_2_O (15 ml) and the aqueous layer was extracted with Et_2_O (20 ml). The combined organic layers were dried (MgSO_4_), filtered, and concentrated *in vacuo*. The crude material was purified by flash column chromatography on silica gel (petroleum ester:EtOAc, 8:1) and recrystallized from EtOAc, yield 3.48 g (78%). Colorless crystals suitable for X-ray diffraction were obtained by recrystallization from a solution of the title compound from ethyl acetate over a period of one week.

### Refinement

H atoms were positioned geometrically and refined using a riding model with C—H = 0.93–0.97 Å, and with *U*_iso_(H) = 1.2*U*_eq_(C).

### Crystal data

**Table:**

C_31_H_30_N_2_O	*F*(000) = 952
*M**_r_* = 446.57	*D*_x_ = 1.261 Mg m^−^^3^
Monoclinic, *P*2_1_/*c*	Mo *K*α radiation, λ = 0.71073 Å
*a* = 10.0270 (11) Å	Cell parameters from 3715 reflections
*b* = 11.2196 (13) Å	θ = 2.1–23.7°
*c* = 21.445 (2) Å	µ = 0.08 mm^−^^1^
β = 102.912 (1)°	*T* = 291 K
*V* = 2351.5 (5) Å^3^	Block, colourless
*Z* = 4	0.26 × 0.22 × 0.20 mm

### Data collection

**Table:**

Bruker SMART APEX CCD diffractometer	5413 independent reflections
Radiation source: sealed tube	4118 reflections with *I* > 2σ(*I*)
Graphite monochromator	*R*_int_ = 0.024
φ and ω scans	θ_max_ = 27.6°, θ_min_ = 2.0°
Absorption correction: multi-scan (*SADABS*; Bruker, 2000)	*h* = −13→11
*T*_min_ = 0.981, *T*_max_ = 0.985	*k* = −14→14
19941 measured reflections	*l* = −27→27

### Refinement

**Table:**

Refinement on *F*^2^	Secondary atom site location: difference Fourier map
Least-squares matrix: full	Hydrogen site location: inferred from neighbouring sites
*R*[*F*^2^ > 2σ(*F*^2^)] = 0.046	H-atom parameters constrained
*wR*(*F*^2^) = 0.128	*w* = 1/[σ^2^(*F*_o_^2^) + (0.0539*P*)^2^ + 0.5346*P*] where *P* = (*F*_o_^2^ + 2*F*_c_^2^)/3
*S* = 1.04	(Δ/σ)_max_ < 0.001
5413 reflections	Δρ_max_ = 0.23 e Å^−^^3^
308 parameters	Δρ_min_ = −0.18 e Å^−^^3^
0 restraints	Extinction correction: *SHELXL97* (Sheldrick, 2008), Fc^*^=kFc[1+0.001xFc^2^λ^3^/sin(2θ)]^-1/4^
Primary atom site location: structure-invariant direct methods	Extinction coefficient: 0.0045 (9)

### Special details

**Table:**

Geometry. All e.s.d.'s (except the e.s.d. in the dihedral angle between two l.s. planes) are estimated using the full covariance matrix. The cell e.s.d.'s are taken into account individually in the estimation of e.s.d.'s in distances, angles and torsion angles; correlations between e.s.d.'s in cell parameters are only used when they are defined by crystal symmetry. An approximate (isotropic) treatment of cell e.s.d.'s is used for estimating e.s.d.'s involving l.s. planes.

### Fractional atomic coordinates and isotropic or equivalent isotropic displacement parameters (Å^2^)

**Table:**

	*x*	*y*	*z*	*U*_iso_*/*U*_eq_	
C1	0.31111 (17)	0.90193 (14)	0.37306 (8)	0.0602 (4)	
H1	0.2667	0.8293	0.3738	0.072*	
C2	0.24286 (19)	0.99559 (15)	0.33757 (9)	0.0678 (5)	
H2	0.1537	0.9853	0.3140	0.081*	
C3	0.3070 (2)	1.10362 (15)	0.33714 (8)	0.0669 (5)	
H3	0.2611	1.1668	0.3136	0.080*	
C4	0.4377 (2)	1.11816 (15)	0.37124 (8)	0.0663 (5)	
H4	0.4807	1.1916	0.3711	0.080*	
C5	0.50674 (18)	1.02485 (14)	0.40593 (7)	0.0571 (4)	
H5	0.5964	1.0357	0.4286	0.069*	
C6	0.44428 (15)	0.91508 (12)	0.40739 (7)	0.0471 (3)	
C7	0.52446 (15)	0.81721 (13)	0.44731 (7)	0.0516 (4)	
H7A	0.6166	0.8170	0.4402	0.062*	
H7B	0.5310	0.8355	0.4921	0.062*	
C8	0.47447 (14)	0.63044 (12)	0.37700 (6)	0.0419 (3)	
H8	0.4309	0.6764	0.3391	0.050*	
C9	0.62199 (15)	0.60282 (15)	0.37396 (7)	0.0547 (4)	
H9A	0.6612	0.5507	0.4094	0.066*	
H9B	0.6738	0.6766	0.3799	0.066*	
C10	0.64000 (18)	0.54450 (16)	0.31205 (8)	0.0635 (4)	
H10A	0.7367	0.5447	0.3121	0.076*	
H10B	0.5941	0.5937	0.2765	0.076*	
C11	0.5869 (2)	0.41688 (16)	0.29973 (9)	0.0690 (5)	
H11A	0.6509	0.3730	0.2806	0.083*	
H11B	0.5875	0.3801	0.3407	0.083*	
C12	0.4439 (2)	0.40138 (15)	0.25704 (8)	0.0683 (5)	
H12A	0.4540	0.3974	0.2131	0.082*	
H12B	0.4097	0.3245	0.2670	0.082*	
C13	0.33303 (18)	0.49374 (14)	0.25950 (7)	0.0580 (4)	
H13A	0.2574	0.4798	0.2232	0.070*	
H13B	0.3699	0.5719	0.2538	0.070*	
C14	0.27571 (15)	0.49744 (12)	0.31984 (6)	0.0460 (3)	
H14	0.2353	0.4191	0.3239	0.055*	
C15	0.38769 (13)	0.51737 (11)	0.38128 (6)	0.0391 (3)	
H15	0.4489	0.4482	0.3872	0.047*	
C16	0.33156 (13)	0.53197 (12)	0.44200 (6)	0.0406 (3)	
H16	0.3689	0.4681	0.4720	0.049*	
C17	0.38781 (13)	0.65000 (13)	0.47065 (6)	0.0450 (3)	
C18	0.17476 (13)	0.53146 (13)	0.43172 (7)	0.0453 (3)	
H18	0.1529	0.5725	0.4685	0.054*	
C19	0.11444 (14)	0.60591 (13)	0.37323 (8)	0.0507 (4)	
C20	0.16338 (15)	0.58891 (13)	0.31758 (7)	0.0507 (4)	
C21	0.10974 (18)	0.65907 (16)	0.26432 (9)	0.0703 (5)	
H21	0.1408	0.6488	0.2269	0.084*	
C22	0.0109 (2)	0.74376 (19)	0.26639 (13)	0.0890 (7)	
H22	−0.0241	0.7898	0.2304	0.107*	
C23	−0.03587 (19)	0.76050 (17)	0.32073 (14)	0.0881 (7)	
H23	−0.1027	0.8176	0.3216	0.106*	
C24	0.01611 (16)	0.69246 (15)	0.37492 (10)	0.0688 (5)	
H24	−0.0148	0.7048	0.4122	0.083*	
C25	0.11027 (13)	0.40779 (13)	0.42908 (6)	0.0441 (3)	
C26	0.18459 (16)	0.30341 (14)	0.43995 (9)	0.0601 (4)	
H26	0.2795	0.3070	0.4474	0.072*	
C27	0.12197 (17)	0.19377 (15)	0.44006 (9)	0.0638 (4)	
H27	0.1746	0.1249	0.4480	0.077*	
C28	−0.01900 (16)	0.18671 (15)	0.42841 (7)	0.0542 (4)	
C29	−0.09470 (17)	0.28951 (17)	0.41747 (10)	0.0696 (5)	
H29	−0.1896	0.2857	0.4097	0.083*	
C30	−0.03091 (16)	0.39835 (16)	0.41795 (9)	0.0640 (4)	
H30	−0.0838	0.4672	0.4106	0.077*	
C31	−0.08463 (18)	0.07266 (18)	0.42638 (9)	0.0673 (5)	
N1	0.46710 (11)	0.69837 (10)	0.43443 (5)	0.0433 (3)	
N2	−0.13830 (19)	−0.01797 (17)	0.42320 (10)	0.0922 (6)	
O1	0.36647 (12)	0.69285 (11)	0.52002 (5)	0.0644 (3)	

### Atomic displacement parameters (Å^2^)

**Table:**

	*U*^11^	*U*^22^	*U*^33^	*U*^12^	*U*^13^	*U*^23^
C1	0.0559 (9)	0.0417 (8)	0.0776 (11)	−0.0027 (7)	0.0031 (8)	0.0007 (7)
C2	0.0631 (10)	0.0535 (10)	0.0794 (12)	0.0063 (8)	−0.0001 (9)	0.0033 (8)
C3	0.0878 (13)	0.0482 (9)	0.0634 (10)	0.0093 (9)	0.0141 (9)	0.0054 (7)
C4	0.0908 (13)	0.0451 (9)	0.0639 (10)	−0.0122 (9)	0.0191 (9)	0.0016 (7)
C5	0.0640 (10)	0.0516 (9)	0.0544 (8)	−0.0130 (7)	0.0106 (7)	−0.0045 (7)
C6	0.0526 (8)	0.0411 (7)	0.0471 (7)	−0.0036 (6)	0.0102 (6)	−0.0070 (6)
C7	0.0495 (8)	0.0442 (8)	0.0555 (8)	−0.0061 (6)	−0.0004 (6)	−0.0057 (6)
C8	0.0455 (7)	0.0393 (7)	0.0382 (6)	−0.0019 (5)	0.0038 (5)	0.0030 (5)
C9	0.0480 (8)	0.0619 (10)	0.0555 (8)	−0.0054 (7)	0.0146 (7)	0.0011 (7)
C10	0.0635 (10)	0.0703 (11)	0.0631 (10)	−0.0019 (8)	0.0278 (8)	0.0019 (8)
C11	0.0844 (13)	0.0600 (10)	0.0724 (11)	0.0088 (9)	0.0387 (10)	−0.0005 (8)
C12	0.1055 (15)	0.0502 (9)	0.0566 (9)	−0.0090 (9)	0.0340 (10)	−0.0083 (7)
C13	0.0795 (11)	0.0511 (9)	0.0393 (7)	−0.0151 (8)	0.0047 (7)	−0.0010 (6)
C14	0.0545 (8)	0.0376 (7)	0.0409 (7)	−0.0072 (6)	0.0003 (6)	0.0021 (5)
C15	0.0404 (7)	0.0355 (6)	0.0392 (6)	0.0019 (5)	0.0043 (5)	0.0033 (5)
C16	0.0365 (6)	0.0431 (7)	0.0394 (6)	0.0023 (5)	0.0027 (5)	0.0048 (5)
C17	0.0376 (7)	0.0508 (8)	0.0425 (7)	0.0027 (6)	0.0004 (5)	−0.0019 (6)
C18	0.0375 (7)	0.0472 (8)	0.0497 (7)	0.0024 (6)	0.0069 (6)	−0.0027 (6)
C19	0.0364 (7)	0.0407 (7)	0.0673 (9)	0.0001 (6)	−0.0047 (6)	0.0016 (6)
C20	0.0464 (8)	0.0426 (8)	0.0527 (8)	−0.0058 (6)	−0.0113 (6)	0.0043 (6)
C21	0.0655 (10)	0.0621 (10)	0.0660 (10)	−0.0076 (8)	−0.0222 (8)	0.0158 (8)
C22	0.0660 (12)	0.0685 (13)	0.1081 (17)	0.0023 (10)	−0.0324 (12)	0.0303 (12)
C23	0.0484 (10)	0.0549 (11)	0.143 (2)	0.0107 (8)	−0.0176 (12)	0.0156 (12)
C24	0.0421 (8)	0.0520 (9)	0.1047 (14)	0.0058 (7)	0.0003 (8)	0.0016 (9)
C25	0.0382 (7)	0.0522 (8)	0.0423 (7)	0.0001 (6)	0.0096 (5)	0.0011 (6)
C26	0.0398 (8)	0.0537 (9)	0.0840 (11)	−0.0010 (7)	0.0076 (7)	0.0076 (8)
C27	0.0518 (9)	0.0540 (10)	0.0843 (12)	0.0005 (7)	0.0126 (8)	0.0082 (8)
C28	0.0518 (9)	0.0606 (10)	0.0515 (8)	−0.0116 (7)	0.0146 (7)	0.0023 (7)
C29	0.0383 (8)	0.0788 (12)	0.0914 (13)	−0.0076 (8)	0.0141 (8)	0.0062 (10)
C30	0.0410 (8)	0.0610 (10)	0.0895 (12)	0.0039 (7)	0.0136 (8)	0.0065 (9)
C31	0.0589 (10)	0.0736 (12)	0.0691 (11)	−0.0160 (9)	0.0136 (8)	0.0043 (9)
N1	0.0432 (6)	0.0403 (6)	0.0433 (6)	−0.0021 (5)	0.0033 (5)	−0.0012 (5)
N2	0.0827 (12)	0.0817 (12)	0.1097 (14)	−0.0295 (10)	0.0162 (10)	0.0052 (10)
O1	0.0637 (7)	0.0772 (8)	0.0538 (6)	−0.0109 (6)	0.0160 (5)	−0.0209 (6)

### Geometric parameters (Å, º)

**Table:**

C1—C6	1.381 (2)	C14—C15	1.5445 (18)
C1—C2	1.385 (2)	C14—H14	0.9800
C1—H1	0.9300	C15—C16	1.5384 (18)
C2—C3	1.373 (2)	C15—H15	0.9800
C2—H2	0.9300	C16—C17	1.5147 (19)
C3—C4	1.360 (3)	C16—C18	1.5380 (18)
C3—H3	0.9300	C16—H16	0.9800
C4—C5	1.378 (2)	C17—O1	1.2243 (17)
C4—H4	0.9300	C17—N1	1.3438 (18)
C5—C6	1.385 (2)	C18—C19	1.516 (2)
C5—H5	0.9300	C18—C25	1.527 (2)
C6—C7	1.509 (2)	C18—H18	0.9800
C7—N1	1.4539 (17)	C19—C24	1.390 (2)
C7—H7A	0.9700	C19—C20	1.401 (2)
C7—H7B	0.9700	C20—C21	1.392 (2)
C8—N1	1.4642 (17)	C21—C22	1.381 (3)
C8—C9	1.527 (2)	C21—H21	0.9300
C8—C15	1.5526 (18)	C22—C23	1.363 (3)
C8—H8	0.9800	C22—H22	0.9300
C9—C10	1.526 (2)	C23—C24	1.391 (3)
C9—H9A	0.9700	C23—H23	0.9300
C9—H9B	0.9700	C24—H24	0.9300
C10—C11	1.530 (2)	C25—C26	1.380 (2)
C10—H10A	0.9700	C25—C30	1.386 (2)
C10—H10B	0.9700	C26—C27	1.381 (2)
C11—C12	1.528 (3)	C26—H26	0.9300
C11—H11A	0.9700	C27—C28	1.382 (2)
C11—H11B	0.9700	C27—H27	0.9300
C12—C13	1.530 (2)	C28—C29	1.372 (2)
C12—H12A	0.9700	C28—C31	1.435 (2)
C12—H12B	0.9700	C29—C30	1.378 (2)
C13—C14	1.529 (2)	C29—H29	0.9300
C13—H13A	0.9700	C30—H30	0.9300
C13—H13B	0.9700	C31—N2	1.145 (2)
C14—C20	1.517 (2)		
			
C6—C1—C2	120.80 (15)	C13—C14—H14	106.9
C6—C1—H1	119.6	C15—C14—H14	106.9
C2—C1—H1	119.6	C16—C15—C14	113.81 (11)
C3—C2—C1	119.92 (17)	C16—C15—C8	106.19 (10)
C3—C2—H2	120.0	C14—C15—C8	112.22 (10)
C1—C2—H2	120.0	C16—C15—H15	108.1
C4—C3—C2	119.90 (16)	C14—C15—H15	108.1
C4—C3—H3	120.1	C8—C15—H15	108.1
C2—C3—H3	120.1	C17—C16—C18	109.72 (11)
C3—C4—C5	120.45 (16)	C17—C16—C15	105.24 (11)
C3—C4—H4	119.8	C18—C16—C15	115.71 (11)
C5—C4—H4	119.8	C17—C16—H16	108.6
C4—C5—C6	120.82 (16)	C18—C16—H16	108.6
C4—C5—H5	119.6	C15—C16—H16	108.6
C6—C5—H5	119.6	O1—C17—N1	125.56 (14)
C1—C6—C5	118.10 (14)	O1—C17—C16	124.95 (13)
C1—C6—C7	123.63 (13)	N1—C17—C16	109.47 (12)
C5—C6—C7	118.26 (13)	C19—C18—C25	112.51 (11)
N1—C7—C6	114.93 (11)	C19—C18—C16	108.83 (11)
N1—C7—H7A	108.5	C25—C18—C16	114.81 (11)
C6—C7—H7A	108.5	C19—C18—H18	106.7
N1—C7—H7B	108.5	C25—C18—H18	106.7
C6—C7—H7B	108.5	C16—C18—H18	106.7
H7A—C7—H7B	107.5	C24—C19—C20	120.18 (15)
N1—C8—C9	111.91 (11)	C24—C19—C18	121.17 (16)
N1—C8—C15	103.97 (10)	C20—C19—C18	118.61 (12)
C9—C8—C15	113.44 (12)	C21—C20—C19	118.50 (16)
N1—C8—H8	109.1	C21—C20—C14	123.84 (16)
C9—C8—H8	109.1	C19—C20—C14	117.64 (12)
C15—C8—H8	109.1	C22—C21—C20	120.7 (2)
C8—C9—C10	115.35 (13)	C22—C21—H21	119.6
C8—C9—H9A	108.4	C20—C21—H21	119.6
C10—C9—H9A	108.4	C23—C22—C21	120.58 (18)
C8—C9—H9B	108.4	C23—C22—H22	119.7
C10—C9—H9B	108.4	C21—C22—H22	119.7
H9A—C9—H9B	107.5	C22—C23—C24	120.17 (19)
C9—C10—C11	116.56 (14)	C22—C23—H23	119.9
C9—C10—H10A	108.2	C24—C23—H23	119.9
C11—C10—H10A	108.2	C23—C24—C19	119.8 (2)
C9—C10—H10B	108.2	C23—C24—H24	120.1
C11—C10—H10B	108.2	C19—C24—H24	120.1
H10A—C10—H10B	107.3	C26—C25—C30	117.17 (14)
C12—C11—C10	116.98 (15)	C26—C25—C18	123.84 (12)
C12—C11—H11A	108.1	C30—C25—C18	118.93 (13)
C10—C11—H11A	108.1	C25—C26—C27	121.84 (14)
C12—C11—H11B	108.1	C25—C26—H26	119.1
C10—C11—H11B	108.1	C27—C26—H26	119.1
H11A—C11—H11B	107.3	C28—C27—C26	119.86 (16)
C13—C12—C11	119.60 (14)	C28—C27—H27	120.1
C13—C12—H12A	107.4	C26—C27—H27	120.1
C11—C12—H12A	107.4	C29—C28—C27	119.17 (15)
C13—C12—H12B	107.4	C29—C28—C31	120.80 (15)
C11—C12—H12B	107.4	C27—C28—C31	120.02 (16)
H12A—C12—H12B	107.0	C28—C29—C30	120.38 (15)
C12—C13—C14	117.47 (13)	C28—C29—H29	119.8
C12—C13—H13A	107.9	C30—C29—H29	119.8
C14—C13—H13A	107.9	C29—C30—C25	121.58 (16)
C12—C13—H13B	107.9	C29—C30—H30	119.2
C14—C13—H13B	107.9	C25—C30—H30	119.2
H13A—C13—H13B	107.2	N2—C31—C28	178.3 (2)
C20—C14—C13	114.09 (12)	C17—N1—C7	121.37 (12)
C20—C14—C15	108.81 (11)	C17—N1—C8	115.06 (11)
C13—C14—C15	112.75 (12)	C7—N1—C8	123.02 (11)
C20—C14—H14	106.9		
			
C6—C1—C2—C3	−1.1 (3)	C16—C18—C19—C20	46.38 (17)
C1—C2—C3—C4	0.4 (3)	C24—C19—C20—C21	−1.0 (2)
C2—C3—C4—C5	0.5 (3)	C18—C19—C20—C21	−178.74 (13)
C3—C4—C5—C6	−0.7 (3)	C24—C19—C20—C14	176.99 (13)
C2—C1—C6—C5	0.8 (2)	C18—C19—C20—C14	−0.76 (19)
C2—C1—C6—C7	179.66 (16)	C13—C14—C20—C21	3.8 (2)
C4—C5—C6—C1	0.1 (2)	C15—C14—C20—C21	130.65 (14)
C4—C5—C6—C7	−178.82 (15)	C13—C14—C20—C19	−174.06 (12)
C1—C6—C7—N1	15.7 (2)	C15—C14—C20—C19	−47.21 (16)
C5—C6—C7—N1	−165.47 (13)	C19—C20—C21—C22	0.2 (2)
N1—C8—C9—C10	−173.41 (12)	C14—C20—C21—C22	−177.63 (15)
C15—C8—C9—C10	69.34 (16)	C20—C21—C22—C23	0.2 (3)
C8—C9—C10—C11	−68.4 (2)	C21—C22—C23—C24	0.2 (3)
C9—C10—C11—C12	96.45 (19)	C22—C23—C24—C19	−1.0 (3)
C10—C11—C12—C13	−34.8 (2)	C20—C19—C24—C23	1.4 (2)
C11—C12—C13—C14	−70.43 (19)	C18—C19—C24—C23	179.09 (15)
C12—C13—C14—C20	−178.28 (13)	C19—C18—C25—C26	130.18 (15)
C12—C13—C14—C15	56.94 (17)	C16—C18—C25—C26	5.0 (2)
C20—C14—C15—C16	47.53 (15)	C19—C18—C25—C30	−52.81 (18)
C13—C14—C15—C16	175.15 (11)	C16—C18—C25—C30	−178.00 (13)
C20—C14—C15—C8	−73.10 (14)	C30—C25—C26—C27	−0.2 (2)
C13—C14—C15—C8	54.52 (15)	C18—C25—C26—C27	176.86 (15)
N1—C8—C15—C16	1.84 (12)	C25—C26—C27—C28	0.6 (3)
C9—C8—C15—C16	123.64 (12)	C26—C27—C28—C29	−0.6 (3)
N1—C8—C15—C14	126.78 (11)	C26—C27—C28—C31	177.93 (16)
C9—C8—C15—C14	−111.42 (13)	C27—C28—C29—C30	0.1 (3)
C14—C15—C16—C17	−124.53 (11)	C31—C28—C29—C30	−178.38 (17)
C8—C15—C16—C17	−0.57 (13)	C28—C29—C30—C25	0.3 (3)
C14—C15—C16—C18	−3.23 (16)	C26—C25—C30—C29	−0.3 (3)
C8—C15—C16—C18	120.72 (12)	C18—C25—C30—C29	−177.49 (16)
C18—C16—C17—O1	55.37 (17)	O1—C17—N1—C7	−7.3 (2)
C15—C16—C17—O1	−179.50 (13)	C16—C17—N1—C7	174.23 (11)
C18—C16—C17—N1	−126.19 (12)	O1—C17—N1—C8	−179.09 (13)
C15—C16—C17—N1	−1.06 (14)	C16—C17—N1—C8	2.48 (15)
C17—C16—C18—C19	76.27 (14)	C6—C7—N1—C17	−96.92 (16)
C15—C16—C18—C19	−42.59 (15)	C6—C7—N1—C8	74.16 (17)
C17—C16—C18—C25	−156.63 (11)	C9—C8—N1—C17	−125.55 (13)
C15—C16—C18—C25	84.51 (14)	C15—C8—N1—C17	−2.75 (14)
C25—C18—C19—C24	100.26 (16)	C9—C8—N1—C7	62.85 (16)
C16—C18—C19—C24	−131.34 (14)	C15—C8—N1—C7	−174.34 (11)
C25—C18—C19—C20	−82.02 (16)		

### Hydrogen-bond geometry (Å, º)

**Table:**

*D*—H···*A*	*D*—H	H···*A*	*D*···*A*	*D*—H···*A*
C29—H29···O1^i^	0.93	2.58	3.301 (2)	134

Symmetry code: (i) −*x*, −*y*+1, −*z*+1.
